# Divide and survive: *PuZFP1* coordinates dual root developmental pathways for drought adaptation in *Populus*.

**DOI:** 10.1093/plphys/kiaf261

**Published:** 2025-06-19

**Authors:** Héctor H Torres-Martínez

**Affiliations:** Assistant Features Editor, Plant Physiology, American Society of Plant Biologists; Department of Biology, Stanford University, Stanford, CA 94305, USA; Howard Hughes Medical Institute, Stanford University, Stanford, CA 94305, USA

Composition of root systems may vary in a species- and environmental context–dependent manner (Ramachandran et al. 2025). Root system architecture (RSA) is shaped throughout a plant's life in response to environmental cues, resulting in a broad diversity of adaptation mechanisms (Viana et al. 2022). Populus species trees develop root systems composed of primary, lateral, and adventitious roots. While much is known about how RSA adapts to external signals in model species like *Arabidopsis thaliana*, the mechanisms used by trees remain less explored.

Despite the economic significance of Populus species, relatively few studies have addressed their intrinsic stress resilience mechanisms. However, evidence does suggest links between RSA and stress resistance. For instance, a basic leucine zipper transcription factor (ZIP1-like) promotes lateral root formation under high osmotic stress (Dash et al. 2017), while MYELOBLASTOSIS ONCOGENE HOMOLOG 40 (MYB40) and WRKY DNA-BINDING PROTEIN 75 (WRKY75) transcriton factors regulate adventitious root development under low phosphorus conditions (Wang et al. 2022). Nonetheless, the regulatory networks orchestrating lateral and adventitious root development in *Populus* under abiotic stress remain largely unknown.

Cys2/His2 (C2H2)-type zinc finger proteins (ZFPs), a family of transcription factors, have been widely implicated in regulating root development and stress responses (Han et al. 2020a). In *A. thaliana*, the transcriptional repressors ARABIDOPSIS ZINC FINGER PROTEIN 2 (AZF2) and SALT TOLERANCE ZINC FINGER PROTEIN (STZ) are expressed predominantly in roots under normal conditions, but their expression is strongly induced by salt, dehydration, and abscisic acid (ABA) treatments. Notably, overexpression of *STZ* leads to inhibited plant growth but increased drought tolerance (Sakamoto et al. 2004). Similarly, inducible overexpression of *AZF1* and *AZF2* downregulates ABA- and osmotic stress–responsive genes, severely limiting plant growth (Kodaira et al. 2011). *AtZP1*, another *A. thaliana* C2H2-type ZFP, is highly expressed in root hairs and negatively regulates basic helix-loop-helix (bHLH) transcription factors involved in root hair initiation and elongation (Han et al. 2020b). Furthermore, expression of *IbZFP1* from drought-tolerant sweet potato in *A. thaliana* improves salt and drought tolerance, with transgenic plants exhibiting enhanced root growth under stress conditions (Wang et al. 2016). Whether C2H2-type ZFPs play a role in shaping RSA to enhance stress tolerance in Populus species has not been addressed until now.

In this issue of *Plant Physiology*, Zhao et al. (2025) reveal that PuZFP1, a C2H2-type ZFP from *Populus ussuriensis*, regulates both lateral and adventitious root development under drought conditions, thus shaping an adaptive RSA (Figure).

**Figure.**
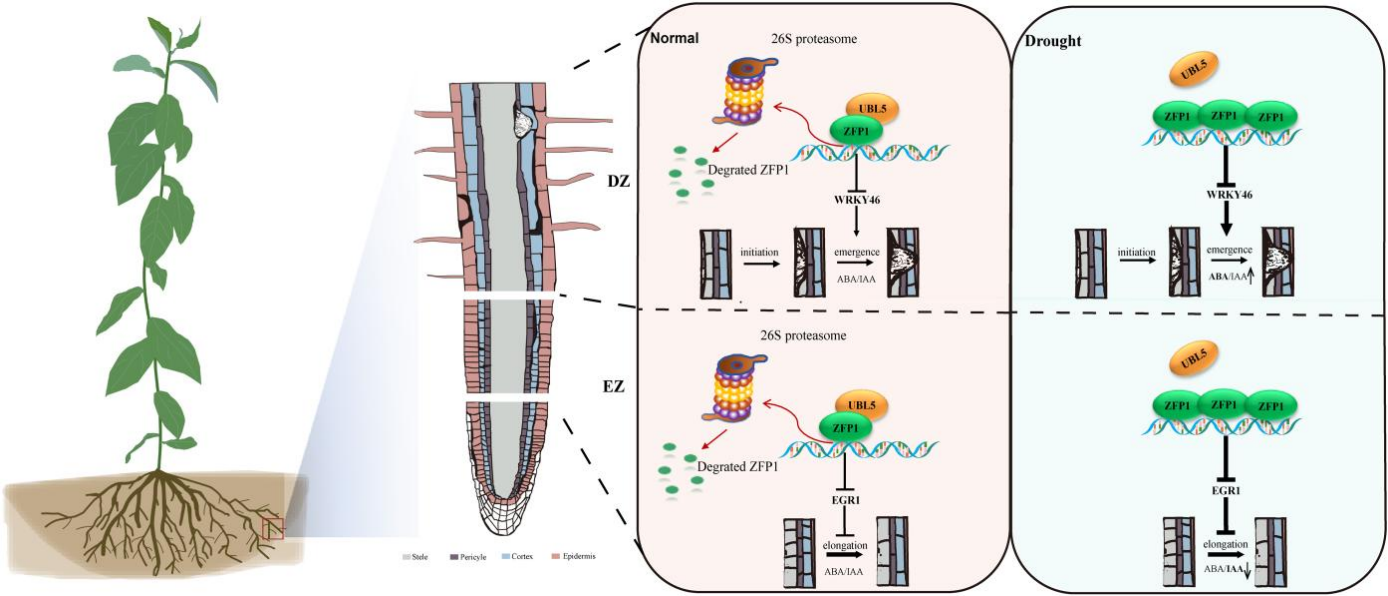
The proposed model of the PuZFP1-PuUBL5 module in *Populus ussuriensis* in response to drought stress. PuUBL5 is accumulated in roots under unstressed conditions. The accumulated PuUBL5 interacts with PuZFP1 and mediates the degradation of PuZFP1 via the 26S protease pathway. However, PuUBL5 expression is inhibited under drought stress, thereby resulting in the accumulation of PuZFP1. The accumulated PuZFP1 is expressed and regulates root development in an auxin-dependent pathway. PuZFP1 inhibits the target gene PuWRKY46 in DZ, thereby inhibiting the emergence of LRP. Meanwhile, PuEGR1 is inhibited in EZ, thereby promoting cell elongation. EZ and DZ denote elongation zone and differentiated zone, respectively (Figure and footnote from Zhao et al. (2025), Fig. 9).

Real-time quantitative PCR (RT-qPCR) showed that *PuZFP1* is upregulated in roots upon drought and ABA treatment. *PuZFP1* promoter-GUS fusion assays confirmed expression in the differentiation zone, and a PuZFP1-GFP translational fusion revealed nuclear and cytoplasmic localization. Transactivation assays using the GAL4-GUS/LUC system demonstrated that PuZFP1 acts as a transcriptional repressor, and mutations in its conserved EAR repression motif abolished this activity.

Functional analyses of transgenic lines revealed the impact of PuZFP1 on root architecture. Under drought, *PuZFP1*-overexpressing lines developed significantly longer adventitious roots but fewer lateral roots. In contrast, RNAi knockdown lines exhibited shorter adventitious roots and increased lateral root formation. Notably, *PuZFP1*-overexpressing plants appeared healthier under drought, whereas wild-type and RNAi lines showed signs of wilting, highlighting PuZFP1's role in drought adaptation.

Yeast 2-hybrid, bimolecular fluorescence complementation, and pull-down assays allowed to identify UBIQUITIN-LIKE PROTEIN 5 (PuUBL5) (Chen et al. 2020) as a direct interactor of PuZFP1. RT-qPCR indicated that *PuUBL5* is highly expressed in roots under nonstressed conditions, but it declined during drought. Protein degradation assays demonstrated that PuUBL5 mediates PuZFP1 turnover via the 26S proteasome, an effect repressed by the proteasome inhibitor MG132. PuZFP1 degradation was accelerated in *PuUBL5*-overexpressing lines, and it was reduced in RNAi lines. Correspondingly, *PuUBL5* overexpression increased lateral root formation, whereas RNAi lines showed enhanced adventitious root development, mirroring the opposite effects of *PuZFP1*.

Transcriptomic analysis of *PuZFP1*-RNAi lines roots, combined with DNA affinity purification sequencing, identified the transcription factors *PuWRKY46* and *PuEGR1* as direct targets of PuZFP1. Consistently, expression analysis of *PuWRKY46* and *PuEGR1* showed that these 2 genes are downregulated in the *PuZFP1*-overexpressing genotype and upregulated in *PuZFP1*-RNAi lines under drought conditions. The regulation of *PuWRKY46* and *PuEGR1* by PuZFP1 was further confirmed by transactivation assays in *Nicotiana benthamiana* leaves through luciferase activity. Overexpression of *PuWRKY46* increased lateral root number, while RNAi lines showed fewer lateral roots. Conversely, *PuEGR1* overexpression promoted longer adventitious roots, with RNAi lines showing the opposite effect.

Spatial expression analysis via RT-qPCR and anatomical observations showed *PuWRKY46* is expressed in the differentiation zone, influencing lateral root primordia development, while *PuEGR1* localizes to the elongation zone, where it regulates cell elongation. Indeed, *PuEGR1*-overexpressing plants exhibited shorter cells in this zone. Interestingly, hormone profiling revealed that in the differentiation zone of *PuZFP1*-overexpressing plants, ABA levels were elevated while indole-3-acetic acid (IAA) was reduced. The opposite pattern was seen in *PuEGR1*-RNAi lines. In the elongation zone, ABA was lower in *PuZFP1*-overexpressing plants whereas it was increased in *PuZFP1-*RNAi plants, with auxin level behaving in the opposite direction (Figure).

In summary, Zhao et al. (2025) uncover a sophisticated regulatory mechanism in which PuZFP1 acts as a drought-responsive transcriptional repressor, modulating distinct aspects of root system architecture through zone-specific gene regulation and hormone balance. PuZFP1 promotes adventitious root elongation while repressing lateral root initiation via *PuEGR1* and *PuWRKY46*, respectively. The regulation of PuZFP1 by PuUBL5 adds another layer of control, linking environmental cues to proteasome-mediated protein turnover (Figure). This study offers critical insights into how trees adapt their RSA under stress, paving the way for genetic strategies to engineer drought-resilient root systems in woody species and beyond.

## Data Availability

No new data were generated or analysed in support of this article.
